# miR-379-5p retards the proliferation and differentiation of goat skeletal muscle satellite cells by targeting *LIN28B*

**DOI:** 10.3389/fvets.2025.1694160

**Published:** 2025-12-04

**Authors:** Wenyue Hou, Shiyu Huang, Zihao Zhao, Xiaoli Xu, Hongping Zhang, Jiazhong Guo, Jiaxue Cao, Dinghui Dai, Li Li

**Affiliations:** College of Animal Science and Technology, Sichuan Agricultural University, Chengdu, China

**Keywords:** goat, MuSCs, miR-379-5p, *LIN28B*, myogenic proliferation and differentiation

## Abstract

MicroRNAs (miRNAs) are emerging as crucial regulators of skeletal muscle development and regeneration; however, the biological functions of many miRNAs remain to be elucidated. In this study, we focused on the function of miR-379-5p, a miRNA we previously identified as highly expressed in the longissimus dorsi muscle of goats. Overexpression of miR-379-5p inhibited the proliferation and differentiation of goat skeletal muscle satellite cells (MuSCs), as evidenced by decreased expression of proliferation and differentiation markers, reduced EdU^+^ cells, and lower myotube formation. Through bioinformatics prediction and experimental validation, we identified *LIN28B* as a direct downstream target of miR-379-5p. Functional assays revealed that *LIN28B* promoted the proliferation and differentiation of MuSCs, whereas miR-379-5p suppressed these processes by decreasing *LIN28B* expression. Furthermore, miR-379-5p inhibited mitochondrial activity during the proliferation phase but promoted it during myogenic differentiation. Additionally, ectopic expression of *LIN28B* decreased mitochondrial membrane potential and enhanced reactive oxygen species (ROS) production, suggesting that *LIN28B* impairs mitochondrial function. Overall, our findings highlight the role of miR-379-5p and *LIN28B* in regulating goat MuSCs activity and mitochondrial function, providing new insights into the role of miRNAs in skeletal muscle development.

## 1 Introduction

Skeletal muscle accounts for approximately 40% of the goat’s body weight and is vital in determining meat yield and quality (1). Skeletal muscle satellite cells (MuSCs) are adult stem cells residing between the basal lamina and the sarcolemma of muscle fibers (2). These cells remain quiescent during homeostasis but are rapidly activated in response to injury or stress, re-entering the cell cycle to initiate muscle regeneration (3, 4). Key regulators of skeletal muscle growth and development include *Pax7* (5), *PCNA* (6), *CCND1* (7), *CDK2* (8), the myogenic regulatory factors *MyoD* (9), *MyoG* (10), and *Myf5* (11), as well as *MyHC* (12) and *MEF2C* (13).

Recent research has unveiled a complex regulatory hierarchy extending beyond protein-coding elements to involve non-coding RNAs (ncRNAs). Among them, microRNAs (miRNAs) are 20 to 25 nucleotides in length (14) and have been implicated in fundamental cellular processes such as proliferation (15), differentiation (16), and apoptosis (17). In the canonical miRNA biogenesis pathway, the Drosha-DGCR8 complex cleaves primary miRNA transcripts into precursor miRNAs, followed by incorporation of the single-stranded miRNA into the RNA-induced silencing complex (RISC), which guides post-transcriptional gene silencing by binding to the 3′ untranslated region (3′ UTR) of target mRNAs through seed-sequence complementarity (18–20). In skeletal muscle, miRNAs have emerged as pivotal regulators of myogenesis that regulate proliferation, differentiation, and regeneration (21). Previous studies have reported that miR-193b-3p (22), miR-145-3p (23), and miR-27b (24) regulate the proliferation and differentiation of MuSCs in goats, underscoring the important roles of miRNAs in goat myogenesis. However, several candidate miRNAs involved in regulating the proliferation and differentiation, particularly those from the imprinted DLK1-DIO3 locus (25), remain insufficiently explored in goats. Notably, miR-379-5p shares an identical mature sequence among goats, mice, and humans, reflecting its conservation across mammals. In mice, miR-379-5p maintains MuSCs in a Pax7^+^ proliferative state and reduces their commitment to myogenic differentiation (26).

Lin28B is a highly conserved RNA-binding protein widely expressed in embryonic stem cells and embryos (27). Conditional deletion of Lin28B in fetal skeletal muscle leads to impaired postnatal growth, indicating that fetal *LIN28B* expression is essential for normal muscle growth (28). In addition, *LIN28B* functions as a crucial post-transcriptional regulator of cellular energy metabolism, modulating the balance between glycolysis and mitochondrial oxidative phosphorylation by regulating the translation of glycolytic and mitochondrial enzymes (29). However, direct evidence of whether *LIN28B* regulates mitochondrial function in MuSCs remains insufficiently explored.

This study mainly used goat tissues and MuSCs to investigate the regulatory axis between miR-379-5p and its predicted target *LIN28B*. Our results demonstrated that miR-379-5p retards MuSC proliferation and differentiation through post-transcriptional regulation of *LIN28B*. Furthermore, we investigated the roles of *LIN28B* and miR-379-5p in regulating mitochondrial membrane potential and reactive oxygen species (ROS) levels in MuSCs. These findings provide new insights into miRNA-mediated regulation of myogenesis and mitochondrial metabolism in goat MuSCs.

## 2 Materials and methods

### 2.1 Animals and sample collection

Newborn Chengdu Ma goats were obtained from the Chengdu Ma Goat Breeding Center (Sichuan, China). All goats were housed in a loose housing barn with slatted floors, allowing free movement and ad libitum access to water. A diet consisting of corn silage, alfalfa hay, and concentrate was offered twice daily at 06:00 and 16:00. Sample collection was preceded by intramuscular administration of Zoletil™ 50 (5.5 mg/kg; Virbac, France) to induce a deep level of anesthesia in the goats. Euthanasia was carried out by trained abattoir personnel via arterial exsanguination in accordance with the Guidelines for Euthanasia of Laboratory Animals issued by the Chinese Association for Laboratory Animal Sciences (T/CALAS 31–2017). Following humane euthanasia, the longissimus dorsi (LD) muscle was aseptically and rapidly collected. Primary MuSCs were then isolated using enzymatic digestion and cultured for subsequent analyses.

### 2.2 Cell isolation

Goat MuSCs were isolated from the LD muscle of Chengdu Ma goats using enzymatic digestion (30). Briefly, muscle tissue was rinsed with sterile phosphate-buffered saline (PBS; Hyclone) to remove blood and debris, trimmed to eliminate connective tissue, and minced into small fragments. The tissue fragments were digested with 0.2% Pronase (Sigma-Aldrich) at 37 °C for 1 h, followed by centrifugation of the suspension at 1500 × g for 6 min to collect the pellet. After washing with PBS, the cells were resuspended in DMEM supplemented with 15% FBS (PAN-Biotech, Germany) and 2% penicillin–streptomycin (Invitrogen, USA), filtered through a 70-μm-mesh sieve (BD Falcon, USA), and centrifuged at 800 × g for 5 min. The isolated cells were subsequently cryopreserved in liquid nitrogen in our laboratory.

### 2.3 Cell culture and transfection

Cells were cultured in T25 or T75 cell culture flasks (Servicebio, China) at 37 °C in a humidified atmosphere containing 5% CO_2_ using a cell incubator (Thermo Fisher, United States). For subsequent experiments, MuSCs were seeded in 6-well, 12-well, or 96-well plates (Servicebio, China). The growth medium (GM) consisted of DMEM supplemented with 10% FBS and 2% penicillin–streptomycin. To induce MuSCs differentiation, the GM was replaced with differentiation medium (DM) containing 2% horse serum (HS; Gibco, United States) and 2% penicillin–streptomycin (Invitrogen, USA) once cells reached 80–90% confluence. Transfections were performed 48 h after proliferation or 24 h after switching to the DM. miR-379-5p mimics or negative control mimics (NC mimics) were synthesized by RiboBio (China). pEGFP-N1 (Tsingke, China) was used to construct the *LIN28B* overexpression plasmid (pLIN28B), with the empty vector (pCtrl) serving as the control. Prior to transfection, cells were incubated with antibiotic-free growth medium. Transfection complexes were prepared by incubating miRNA mimics or plasmids with Lipofectamine™ 3,000 (Invitrogen, United States) in DMEM, following the manufacturer’s protocol. After 4–6 h of transfection, the transfection medium was replaced with antibiotic-free proliferation or differentiation medium. Cells were harvested 48 h post-transfection using appropriate lysis buffers for RNA and protein extraction for downstream analyses.

### 2.4 RNA extraction and cDNA synthesis

Total RNA was extracted from goat MuSCs using the Omega BIO-TEK DNA/RNA/Protein Kit (Omega Bio-tek, United States) according to the manufacturer’s instructions. RNA concentration and quality were measured using a NanoDrop 2000 spectrophotometer (Thermo Fisher Scientific, United States), based on the OD260/OD280 and OD260/OD230. cDNA was synthesized from 1 μg of total RNA using the HiScript III RT SuperMix for qPCR (+gDNA wiper) (Vazyme, China) for mRNA and from 0.5 μg of RNA using the Mir-X miRNA First-Strand Synthesis Kit (Takara Bio, China) for miRNA, following the manufacturers’ instructions.

### 2.5 RT-qPCR analysis

RT-qPCR primers were designed using Primer-BLAST (NCBI) according to standard primer design principles or adopted from published literature. The corresponding primer sequences are provided in Supplementary Table S1. miRNA expression levels were quantified by RT-qPCR using the TB Green Premix Ex Taq II (Tli RNaseH Plus) Kit (Takara, China) on a CFX96 Real-Time PCR Detection System (Bio-Rad, United States). The reaction system contained 9 μL ddH₂O, 12.5 μL TB Green Advantage Premix, 0.5 μL ROX Dye (50×), 0.5 μL miRNA-specific primer (10 μM), 0.5 μL mRQ 3’Primer (10 μM), and 2 μL cDNA. The reaction program included an initial denaturation at 95 °C for 30 s, followed by 39 cycles of denaturation at 95 °C for 5 s and annealing at a specific temperature for 30 s. For mRNA quantification, RT-qPCR was performed with the ChamQ SYBR qPCR Master Mix (Q311-02; Vazyme, China). The reaction system consisted of 3.4 μL ddH₂O, 5 μL ChamQ SYBR qPCR Master Mix, 0.4 μL forward primer, 0.4 μL reverse primer, and 0.8 μL cDNA. The reaction program included an initial denaturation at 95 °C for 5 min, followed by 39 cycles of denaturation at 95 °C for 10 s and annealing at a specific temperature for 30 s. All procedures were conducted in strict accordance with the manufacturers’ protocols.

### 2.6 Western Blot

Western blot was performed using FuturePAG™ precast gels (ACE, China) at 160 V for 30 min. Following transfer, membranes were blocked and incubated with primary antibodies at 4 °C for 16 h. Then, the membranes were washed four times with TBST (1 L: 8.8 g NaCl, 10 mL Tris–HCl, 500 μL Tween-20) for 10 min each. Secondary antibody incubation was carried out at 37 °C for 90 min, followed by four TBST washes (10 min each) and two TBS washes (5 min each). Chemiluminescent detection was performed using BeyoECL Star reagent (Beyotime, China).

Primary antibodies included Pax7 (1:200, Santa Cruz Biotechnology, China), PCNA (1:200, Santa Cruz Biotechnology, China), *β*-Tubulin mouse mAb (1:5000, ZenBioScience, China), and MyoD polyclonal antibody (1:1000, Proteintech, United States). HRP-conjugated goat anti-rabbit IgG (H + L) and goat anti-mouse IgG (H + L) (both 1:10000, ABclonal, China) were used as secondary antibodies.

### 2.7 EdU proliferation assay

The EdU assay was performed using the Cell-Light EdU Apollo567 *in vitro* Kit (100 T; RiboBio, China). Briefly, goat MuSCs cultured in 12-well plates at 48 h post-transfection were incubated with 50 μM EdU working solution (1:1000 dilution in culture medium) for 2 h. After fixation with 4% paraformaldehyde and treatment with 2 mg/mL glycine, cells were washed with PBS and permeabilized with 0.5% Triton X-100 (Coolaber, China). Next, cells were stained with 1 × Apollo staining solution for 30 min, followed by nuclear staining with Hoechst 33342 for 30 min in the dark. After PBS washes, cells were imaged using fluorescence microscopy (Olympus, Japan). At least three images per group were captured. The ratio of EdU^+^ cells was calculated as follows: (EdU^+^ cells/DAPI-stained cells) × 100%.

### 2.8 CCK-8 cell viability assay

Cell suspensions were seeded in 96-well plates, with 5 technical replicates per group, and cultured at 37 °C with 5% CO_2_. At 0, 24, 48, and 72 h post-transfection, fresh medium containing 10% CCK-8 reagent was added by replacing the GM with the CCK-8 solution. After 2-h incubation, absorbance at 450 nm was measured using a MultiskanGO microplate reader (Thermo Fisher, United States). Cell viability in transfection groups was calculated as: absorbance of transfection group - absorbance of blank control.

### 2.9 MyHC immunofluorescence staining

After 24 h of differentiation, cells were transfected with miRNA mimics or plasmids, and MyHC immunofluorescence staining was performed 96 h post-transfection in 6-well plates. Cells were fixed with 4% paraformaldehyde for 15 min, permeabilized with 0.5% Triton X-100 for 10 min, and blocked with 2% bovine serum albumin (BSA) for 30 min. Primary antibody incubation was performed using MYH antibody (1:200, Santa Cruz Biotechnology, China) at 4 °C for 12 h, followed by incubation with Cy3-conjugated secondary antibody (1:200, ABclonal, China) at 37 °C for 2 h. Nuclear staining was performed with DAPI for 10 min, followed by PBS washes. The stained cells were imaged under fluorescence microscopy. At least three independent repeats were performed for each treatment, and three random fields were analyzed for each sample. MyHC^+^ cells were quantified by calculating the percentage of nuclei surrounded by the MyHC signal. The fusion index was calculated as: (number of multinucleated myotubes/total number of MyHC^+^ cells) × 100%.

### 2.10 Prediction of genes targeted by miR-379-5p

To investigate the molecular mechanisms of miR-379-5p during MuSC proliferation and differentiation, potential target genes were predicted using the online platforms TargetScan,^1^ miRDB,^2^ miRmap,^3^ and miRwalk.^4^

### 2.11 Dual-luciferase reporter assay

A dual-luciferase reporter assay was used to investigate the binding interaction between miR-379-5p and *LIN28B*. Wild-type and mutant 3′UTR fragments were amplified and subcloned into the psiCHECK-2 vector (Tsingke, China) and subsequently co-transfected with miR-379-5p mimics or NC mimics. Cells were harvested 48 h post-transfection using lysis buffer (TransGen, China). Luminescence measurements were performed using the TransDetect^®^ Double-Luciferase Reporter Assay Kit (TransGen, China) by sequential addition of reagents: 100 μL firefly luciferase solution was mixed with 20 μL cell lysate for the first detection, followed by addition of 100 μL renilla luciferase solution and mixing before the second detection. The relative luciferase activity was calculated as the ratio of Renilla to firefly luciferase activity.

### 2.12 Mitochondrial ROS and membrane potential detection

The Reactive Oxygen Species Assay Kit (Solarbio, China) was used to measure intracellular ROS levels. The ROS-sensitive probe DCFH-DA was diluted 1:2000 in serum-free medium and added to the transfected cells (1 mL per well). After a 30-min incubation, probe-loaded cells were washed three times with serum-free medium and analyzed by fluorescence microscopy. Mean fluorescence intensity was quantified using Image J software (version 1.54). The JC-1 Mitochondrial Membrane Potential Assay Kit (Solarbio, China) was used according to the manufacturer’s instructions. Transfected cells were washed with PBS, incubated with JC-1 working solution, and incubated at 37 °C for 20 min. The solution was then aspirated, and cells were washed twice with JC-1 buffer before fluorescence microscopy imaging. The JC-1 aggregate-to-monomer fluorescence ratio was quantified using Image J software (version 1.54).

### 2.13 Statistical analysis

Statistical analysis was performed using GraphPad Prism 10.1.2 software (GraphPad Software, San Diego, United States). Unpaired Student’s t-test was used to evaluate the difference between the means of two groups. One-way analysis of variance (ANOVA) was used for comparisons involving three or more groups, with Tukey’s test applied for multiple comparisons. Data are presented as the mean ± standard error of the mean (SEM). Statistical significance was defined as * *p* < 0.05, ** *p* < 0.01, and ns *p* > 0.05.

## 3 Results

### 3.1 miR-379-5p suppresses proliferation and impairs mitochondrial function in proliferating goat MuSCs

**Figure 1:**
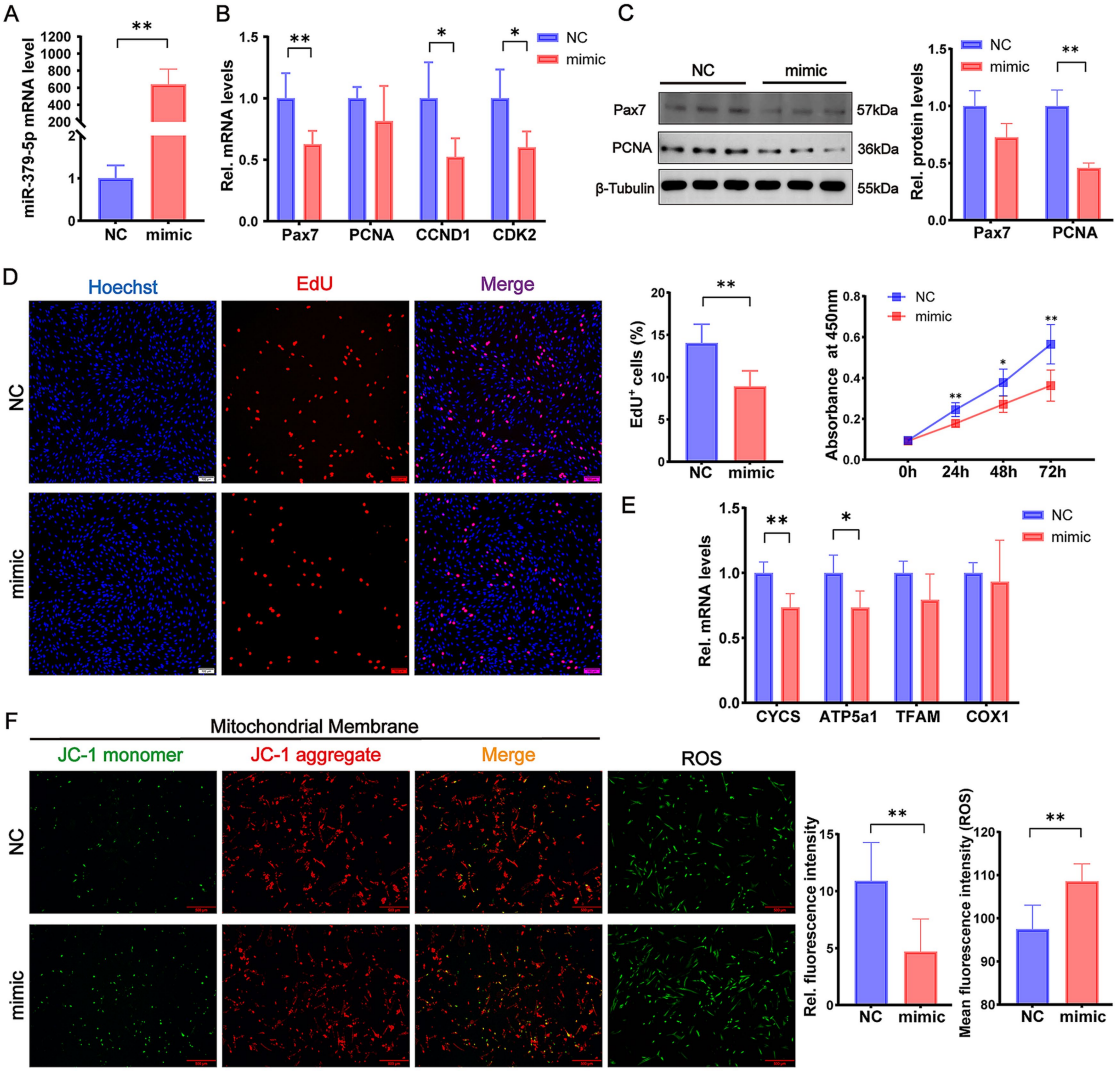
miR-379-5p inhibits proliferation and mitochondrial function in proliferating goat MuSCs. **(A)** Relative expression level of miR-379-5p in goat MuSCs transfected with NC or miR-379-5p mimics during the proliferation phase. **(B)** Relative mRNA levels of proliferation marker genes (*Pax7*, *PCNA*, *CCND1*, and *CDK2*) in MuSCs transfected with NC or miR-379-5p mimics. **(C)** Western blot analysis of Pax7 and PCNA protein levels in proliferating MuSCs transfected with NC or miR-379-5p mimics. *β*-Tubulin was used as a loading control. **(D)** Cell proliferation of goat MuSCs evaluated by EdU incorporation assay (scale bar = 100 μm), with quantification of EdU^+^ cells and CCK-8 assay. **(E)** Relative mRNA levels of mitochondrial marker genes (*CYCS*, *ATP5a1*, *TFAM*, and *COX1*) in proliferating MuSCs transfected with NC or miR-379-5p mimics. **(F)** Representative fluorescence images and quantification of mitochondrial membrane potential and ROS levels in proliferating MuSCs. Left: JC-1 staining reflects mitochondrial membrane potential. ROS levels were indicated by green fluorescence. Middle: Ratio of fluorescence intensity. Right: Quantification of ROS fluorescence intensity. Scale bar = 500 μm. Each experiment contained three biological replicates. * *p* < 0.05, ** *p* < 0.01.

The effect of miR-379-5p on MuSC proliferation was examined by transfecting cells with a miR-379-5p mimic during the proliferation phase. Quantitative analysis confirmed robust overexpression of miR-379-5p in the mimic group, with levels exceeding 400-fold relative to the negative control (NC) group (*p* < 0.01, Figure 1A), indicating high transfection efficiency. Further analysis of proliferation-related gene expression revealed significant downregulation of *Pax7*, *CCND1*, and *CDK2* mRNA levels in the mimic group (*p* < 0.05), whereas *PCNA* mRNA level showed no significant difference (*p* > 0.05, Figure 1B). At the protein level, PCNA was significantly reduced (*p* < 0.01), and Pax7 showed a decreasing trend (*p* = 0.0582, Figure 1C). Functional assays further confirmed these observations: the EdU assay showed a marked reduction in EdU^+^ cells in the mimic group (*p* < 0.01), and the CCK-8 assay revealed significantly reduced cell viability at 24 h, 48 h, and 72 h post-transfection compared with the NC group (*p* < 0.05; Figure 1D). These findings indicate that miR-379-5p overexpression suppresses the proliferation of goat MuSCs.

Subsequent analysis focused on the effects of miR-379-5p on mitochondrial function during MuSC proliferation. We selected *CYCS* (31), *ATP5a1* (32), *COX1* (33), and *TFAM* (34) as representative markers of mitochondrial function, with the former three encoding core components of the respiratory chain that drive electron transfer and ATP synthesis, and the latter regulating mitochondrial DNA replication and transcription. Overexpression of miR-379-5p significantly downregulated the mRNA levels of *CYCS* (*p* < 0.01) and *ATP5a1* (*p* < 0.05; Figure 1E) relative to the NC group. In addition, the JC-1 aggregate-to-monomer fluorescence ratio, an indicator of mitochondrial membrane potential, was significantly reduced in the mimic group (*p* < 0.01; Figure 1F). Consistent with impaired mitochondrial function, the average ROS level was significantly higher in the mimic group than in the NC group (*p* < 0.01; Figure 1F). The concurrent increase in ROS levels and loss of membrane potential highlights their association under oxidative stress, where ROS accumulation promotes depolarization (35, 36). These results suggest that miR-379-5p overexpression not only inhibits MuSC proliferation but also compromises mitochondrial function.

### 3.2 miR-379-5p suppresses differentiation and promotes mitochondrial function in differentiating goat MuSCs

**Figure 2:**
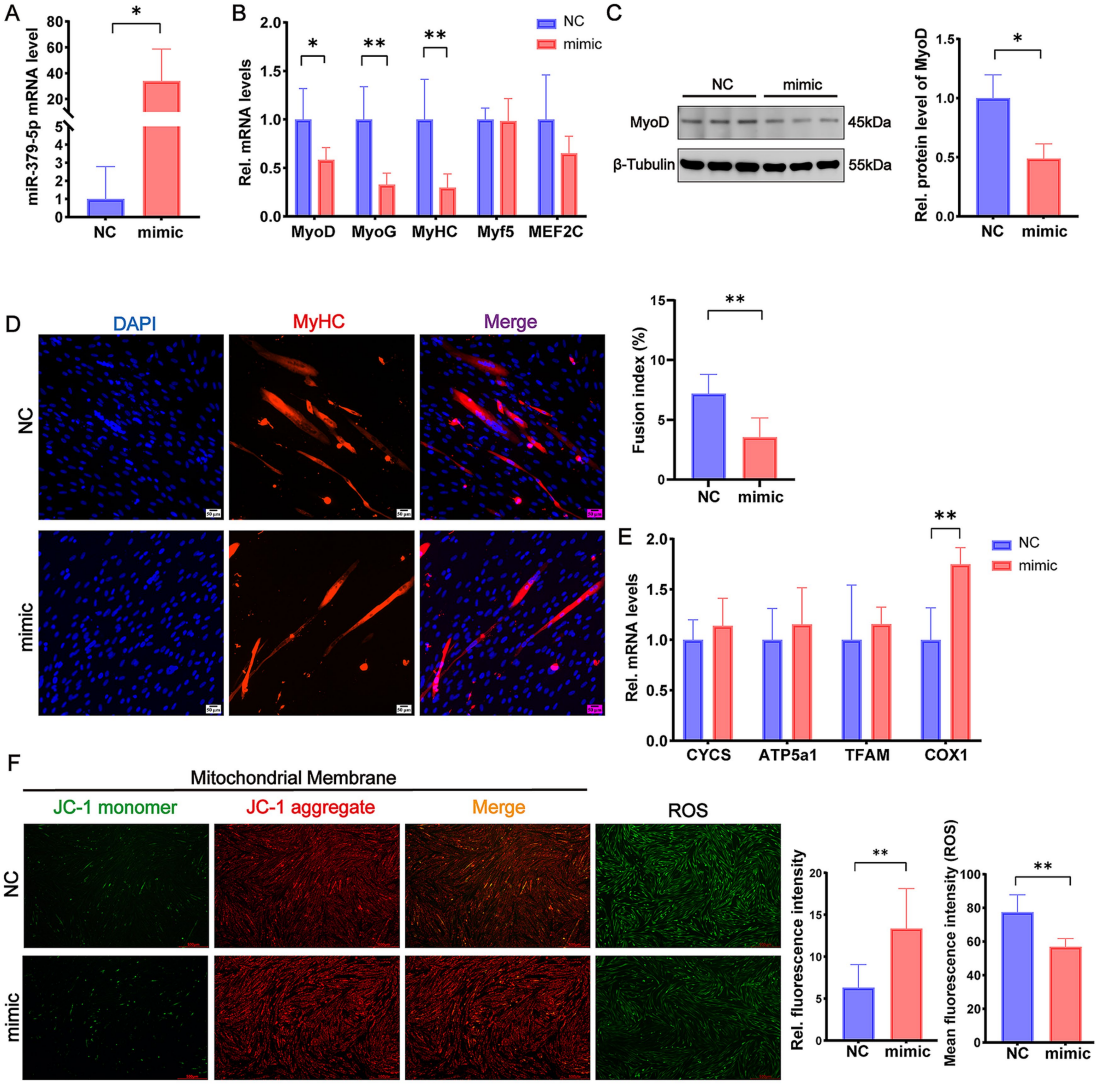
miR-379-5p inhibits differentiation and promotes mitochondrial function in differentiating goat MuSCs. **(A)** Relative expression level of miR-379-5p in goat MuSCs transfected with NC or miR-379-5p mimics during the differentiation phase. **(B)** Relative mRNA levels of differentiation marker genes (*MyoD*, *MyoG*, *MyHC*, *Myf5*, and *MEF2C*) in MuSCs transfected with NC or miR-379-5p mimics. **(C)** Western blot analysis of MyoD protein levels in differentiating MuSCs transfected with NC or miR-379-5p mimics. β-Tubulin was used as a loading control. **(D)** Representative immunofluorescence images of MyHC and DAPI in differentiating goat MuSCs after miR-379-5p overexpression. The right panel shows quantification of the fusion index. Scale bar = 50 μm. **(E)** Relative mRNA levels of mitochondrial marker genes (*CYCS*, *ATP5a1*, *TFAM*, and *COX1*) in differentiating MuSCs transfected with NC or miR-379-5p mimics. **(F)** Representative fluorescence images and quantification of mitochondrial membrane potential and ROS levels in differentiating MuSCs. Left: JC-1 staining reflects mitochondrial membrane potential. ROS levels were indicated by green fluorescence. Middle: Ratio of fluorescence intensity. Right: Quantification of ROS fluorescence intensity. Scale bar = 500 μm. Each experiment contained three biological replicates. * *p* < 0.05, ** *p* < 0.01.

To investigate the role of miR-379-5p during differentiation, goat MuSCs were transfected with a miR-379-5p mimic. Quantitative analysis confirmed that miR-379-5p expression was increased by more than 10-fold in the mimic group compared with the NC group (*p* < 0.05; Figure 2A). This upregulation significantly reduced the mRNA levels of differentiation marker genes, including *MyoD* (*p* < 0.05), *MyoG* (*p* < 0.01), and *MyHC* (*p* < 0.01), whereas *Myf5* and *MEF2C* expression levels remained unchanged (*p* > 0.05; Figure 2B). Western blot analysis further confirmed a significant reduction in MyoD protein levels in the mimic group (*p* < 0.05; Figure 2C). Immunofluorescence staining for MyHC further revealed a decrease in myotube formation, accompanied by a markedly lower fusion index in the mimic group compared with the NC group (*p* < 0.01; Figure 2D). These findings suggest that miR-379-5p suppresses the differentiation of goat MuSCs.

We next assessed whether miR-379-5p affects mitochondrial function during differentiation. Overexpression of miR-379-5p significantly increased the mRNA expression of *COX1* (*p* < 0.01; Figure 2E). Moreover, the JC-1 aggregate-to-monomer fluorescence ratio was significantly elevated in the mimic group, indicating enhanced mitochondrial membrane potential, which was accompanied by a significant reduction in mean ROS levels (*p* < 0.01; Figure 2F). These results indicate that miR-379-5p suppresses differentiation while simultaneously enhancing mitochondrial membrane potential and attenuating oxidative stress in differentiating MuSCs.

### 3.3 miR-379-5p targets and reduces *LIN28B* expression

**Figure 3:**
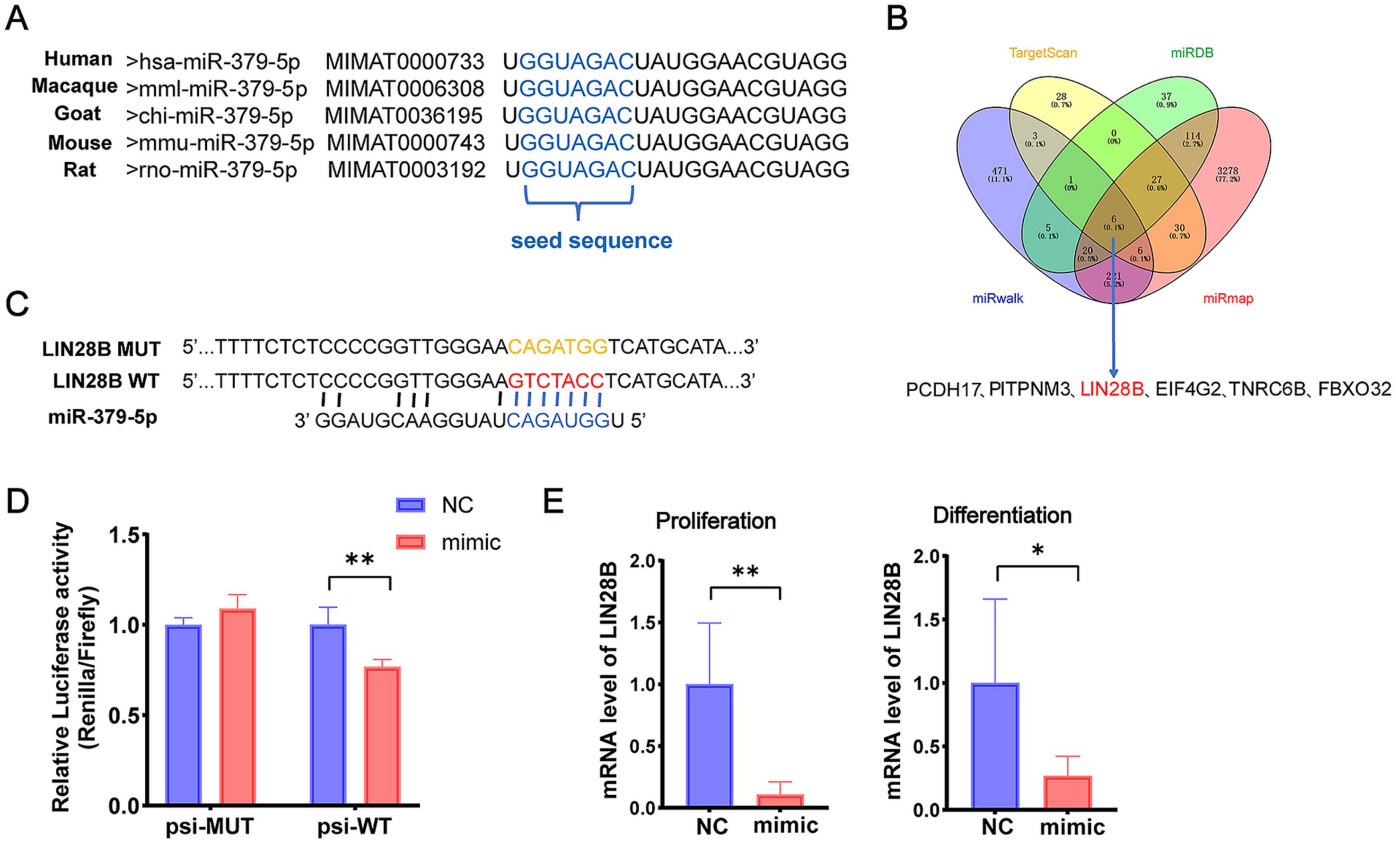
miR-379-5p targets *LIN28B*. **(A)** Sequence comparison of mature miR-379-5p across five species. **(B)** Venn diagram of predicted miR-379-5p target genes. **(C)** Design of psi-WT and psi-MUT *LIN28B* sequences for dual-luciferase reporter assay. **(D)** Relative luciferase activity in cells co-transfected with miR-379-5p mimics and psi-WT or psi-MUT. **(E)** Relative mRNA expression of *LIN28B* in MuSCs following miR-379-5p overexpression during the proliferation and differentiation phases. Each experiment contained three biological replicates. * *p* < 0.05, ** *p* < 0.01.

To explore the molecular mechanism by which miR-379-5p regulates MuSCs, the sequence conservation of miR-379-5p was first analyzed using miRBase.^5^ The mature sequence of goat miR-379-5p was found to be identical across humans, macaques, mice, and rats, indicating a high degree of evolutionary conservation across species (Figure 3A). Furthermore, four online databases (TargetScan, miRDB, miRmap, and miRwalk) were used to predict potential target genes, and six shared candidate targets were identified: *PCDH17*, *PITPNM3*, *LIN28B*, *EIF4G2*, *TNRC6B*, and *FBXO32* (Figure 3B). Among these, *LIN28B* was selected for further investigation due to its reported involvement in mitochondrial oxidative metabolism (29) and regulation of oxidative phosphorylation (OXPHOS) (37).

To validate this interaction, wild-type (psi-WT) and mutant (psi-MUT) fragments of the *LIN28B* 3′UTR were synthesized and co-transfected with miR-379-5p for dual-luciferase reporter assays (Figure 3C). Co-transfection of miR-379-5p with psi-WT significantly reduced relative luciferase activity (*p* < 0.01), whereas no significant change was observed in the psi-MUT group (*p* > 0.05, Figure 3D). In addition, overexpression of miR-379-5p significantly decreased *LIN28B* mRNA levels (*p* < 0.01) in proliferating goat MuSCs, and a similar reduction was observed during the differentiation phase (*p* < 0.05; Figure 3E). Collectively, these findings demonstrate that miR-379-5p directly targets *LIN28B* in goat MuSCs.

### 3.4 *LIN28B* promotes proliferation and impairs mitochondrial function in proliferating goat MuSCs

**Figure 4:**
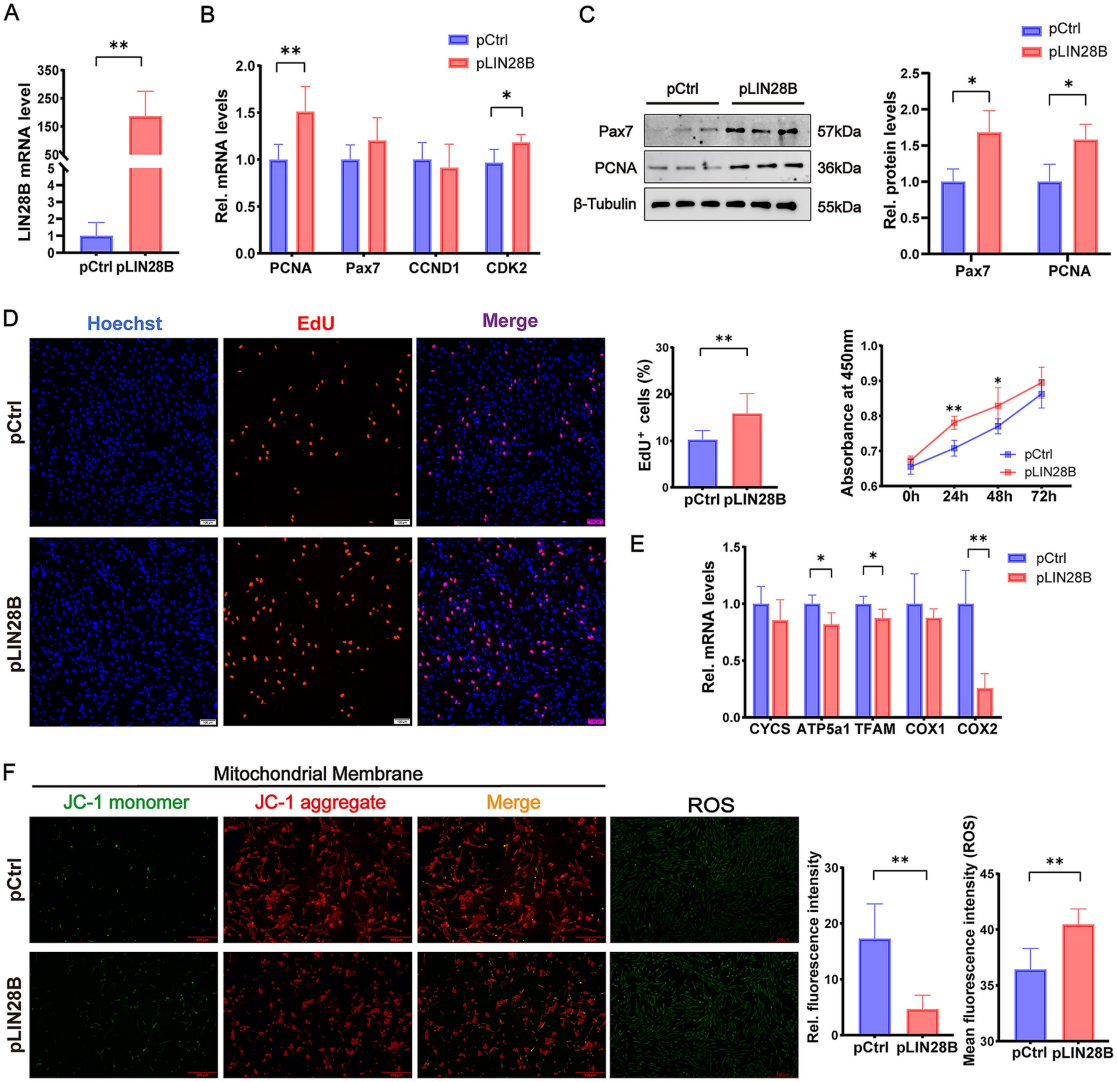
*LIN28B* promotes proliferation and impairs mitochondrial function in proliferating goat MuSCs. **(A)** Relative *LIN28B* mRNA level in goat MuSCs transfected with pCtrl or pLIN28B during the proliferation phase. **(B)** Relative mRNA levels of proliferation marker genes (*PCNA*, *Pax7*, *CCND1*, and *CDK2*) in MuSCs transfected with pCtrl or pLIN28B. **(C)** Western blot analysis of Pax7 and PCNA protein levels in proliferating MuSCs transfected with pCtrl or pLIN28B. β-Tubulin was used as a loading control. **(D)** EdU and CCK-8 assays of goat MuSCs transfected with pCtrl or pLIN28B, including representative EdU immunofluorescence images and EdU^⁺^ cell quantification at 48 h post-transfection, as well as cell viability measured by CCK-8 at 0, 24, 48, and 72 h post-transfection. **(E)** Relative mRNA levels of mitochondrial marker genes (*CYCS*, *ATP5a1*, *TFAM*, *COX1, and COX2*) in proliferating MuSCs transfected with pCtrl or pLIN28B. **(F)** Representative fluorescence images and quantification of mitochondrial membrane potential and ROS levels in proliferating MuSCs. Left: JC-1 staining reflects mitochondrial membrane potential. ROS levels were indicated by green fluorescence. Middle: Ratio of fluorescence intensity. Right: Quantification of ROS fluorescence intensity. Scale bar = 500 μm. Each experiment contained three biological replicates. * *p* < 0.05, ** *p* < 0.01.

To investigate the role of *LIN28B* in MuSC proliferation, proliferating goat MuSCs were transfected with pLIN28B. Quantitative analysis confirmed robust overexpression, with *LIN28B* mRNA levels elevated over 100-fold compared with the pCtrl group (*p* < 0.01; Figure 4A). Correspondingly, the mRNA levels of proliferation markers *PCNA* (*p* < 0.01) and *CDK2* (*p* < 0.05) were significantly upregulated in the pLIN28B group (Figure 4B). Western blot analysis showed significant increases in PCNA and Pax7 expression following *LIN28B* overexpression (*p* < 0.05; Figure 4C). The CCK-8 assay revealed that absorbance values at 24 h (*p* < 0.01) and 48 h (*p* < 0.05) post-transfection were significantly higher relative to the pCtrl group (Figure 4D). Consistently, *LIN28B* overexpression significantly increased the percentage of EdU^+^ cells compared with the pCtrl group (*p* < 0.01; Figure 4D).

Regarding mitochondrial function, *LIN28B* overexpression significantly reduced mRNA levels of *ATP5a1* (*p* < 0.05), *TFAM* (*p* < 0.05), and *COX2* (*p* < 0.01) in proliferating MuSCs (Figure 4E). JC-1 staining further revealed a significant decrease in the aggregate-to-monomer fluorescence ratio (*p* < 0.01), along with increased ROS levels (*p* < 0.01) relative to the pCtrl group (Figure 4F). These results indicate that *LIN28B* enhances MuSC proliferation while impairing mitochondrial function.

### 3.5 *LIN28B* promotes differentiation and impairs mitochondrial function in differentiating goat MuSCs

**Figure 5:**
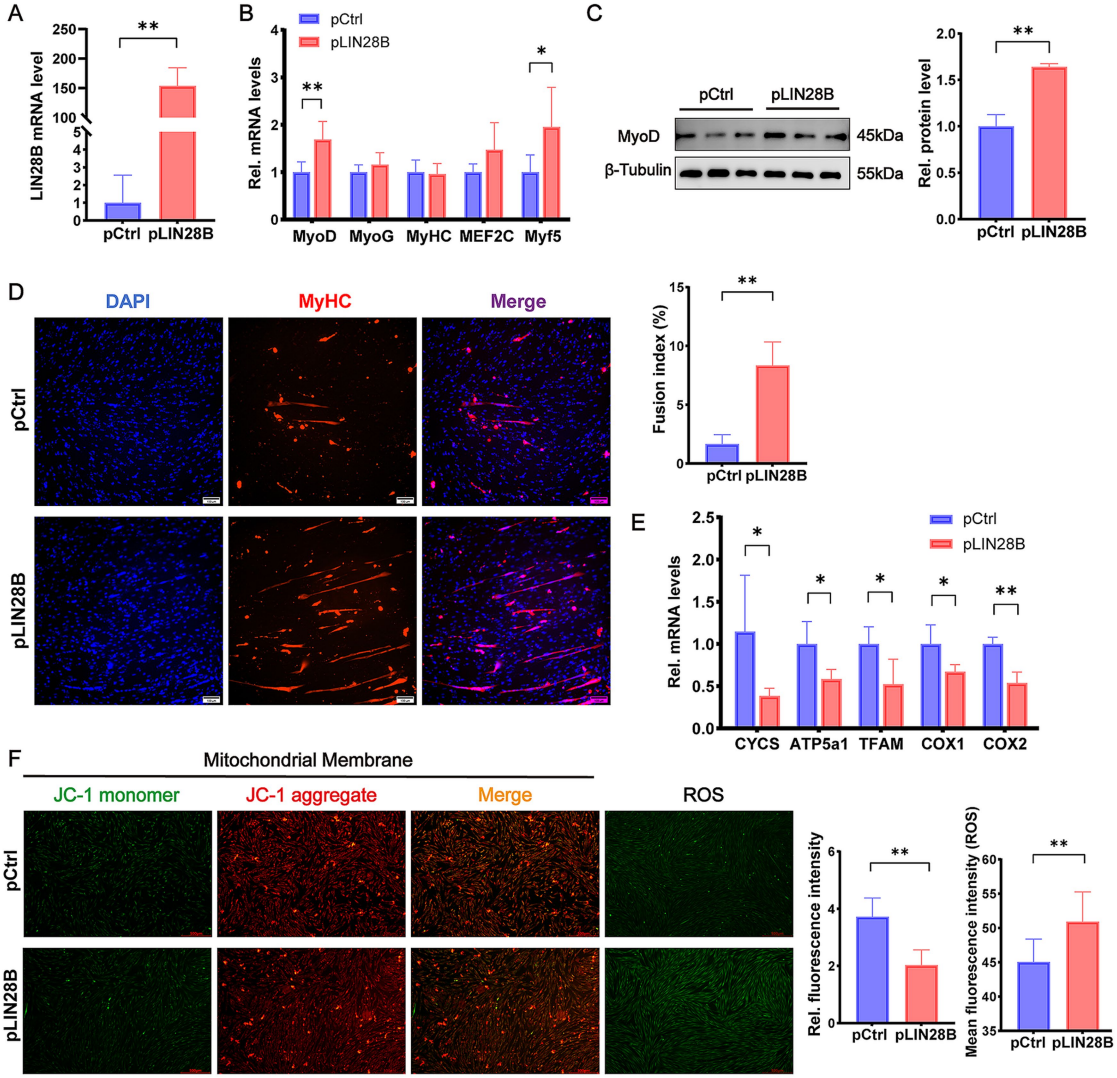
*LIN28B* promotes differentiation and impairs mitochondrial function in differentiating goat MuSCs. **(A)** Relative mRNA level of *LIN28B* in goat MuSCs transfected with pCtrl or pLIN28B during the differentiation phase. **(B)** Relative mRNA levels of differentiation marker genes (*MyoD*, *MyoG*, *MyHC*, *MEF2C*, and *Myf5*) in MuSCs transfected with pCtrl or pLIN28B. **(C)** Western blot analysis of MyoD protein levels in differentiating MuSCs transfected with pCtrl or pLIN28B. β-Tubulin was used as a loading control. **(D)** Representative immunofluorescence images of MyHC and DAPI in differentiating MuSCs transfected with pCtrl or pLIN28B. The right panel shows quantification of the fusion index. Scale bar = 100 μm. **(E)** Relative mRNA levels of mitochondrial marker genes (*CYCS*, *ATP5a1*, *TFAM*, *COX1, and COX2*) in differentiating MuSCs transfected with pCtrl or pLIN28B. **(F)** Representative fluorescence images and quantification of mitochondrial membrane potential and ROS levels in differentiating MuSCs. Left: JC-1 staining reflects mitochondrial membrane potential. ROS levels were indicated by green fluorescence. Middle: Ratio of fluorescence intensity. Right: Quantification of ROS fluorescence intensity. Scale bar = 500 μm. Each experiment contained three biological replicates. * *p* < 0.05, ** *p* < 0.01.

*LIN28B* was overexpressed in differentiating goat MuSCs to investigate its role in differentiation. *LIN28B* mRNA levels increased more than 50-fold in the pLIN28B group relative to the pCtrl group (Figure 5A). RT-qPCR analysis revealed that *LIN28B* overexpression significantly increased the mRNA levels of the myogenic differentiation markers *MyoD* (*p* < 0.01) and *Myf5* (*p* < 0.05), as shown in Figure 5B. Western blot analysis further confirmed a significant elevation of MyoD protein levels (*p* < 0.01) in the pLIN28B group (Figure 5C). Immunofluorescence staining of MyHC showed a significantly increased fusion index (*p* < 0.01) in *LIN28B*-overexpressing cells relative to the pCtrl group (Figure 5D). Collectively, these results indicate that *LIN28B* overexpression enhances the differentiation of goat MuSCs.

In contrast, *LIN28B* overexpression impaired mitochondrial function, as evidenced by the significant downregulation of *CYCS* (*p* < 0.05), *ATP5a1* (*p* < 0.05), *TFAM* (*p* < 0.05), *COX1* (*p* < 0.05), and *COX2* (*p* < 0.01) mRNA levels (Figure 5E). *LIN28B* overexpression resulted in a significantly lower JC-1 aggregate-to-monomer fluorescence ratio (*p* < 0.01) in JC-1–stained cells and higher ROS production (*p* < 0.01), reflecting mitochondrial dysfunction (Figure 5F).

### 3.6 miR-379-5p inhibits proliferation of goat MuSCs by regulating *LIN28B*

**Figure 6:**
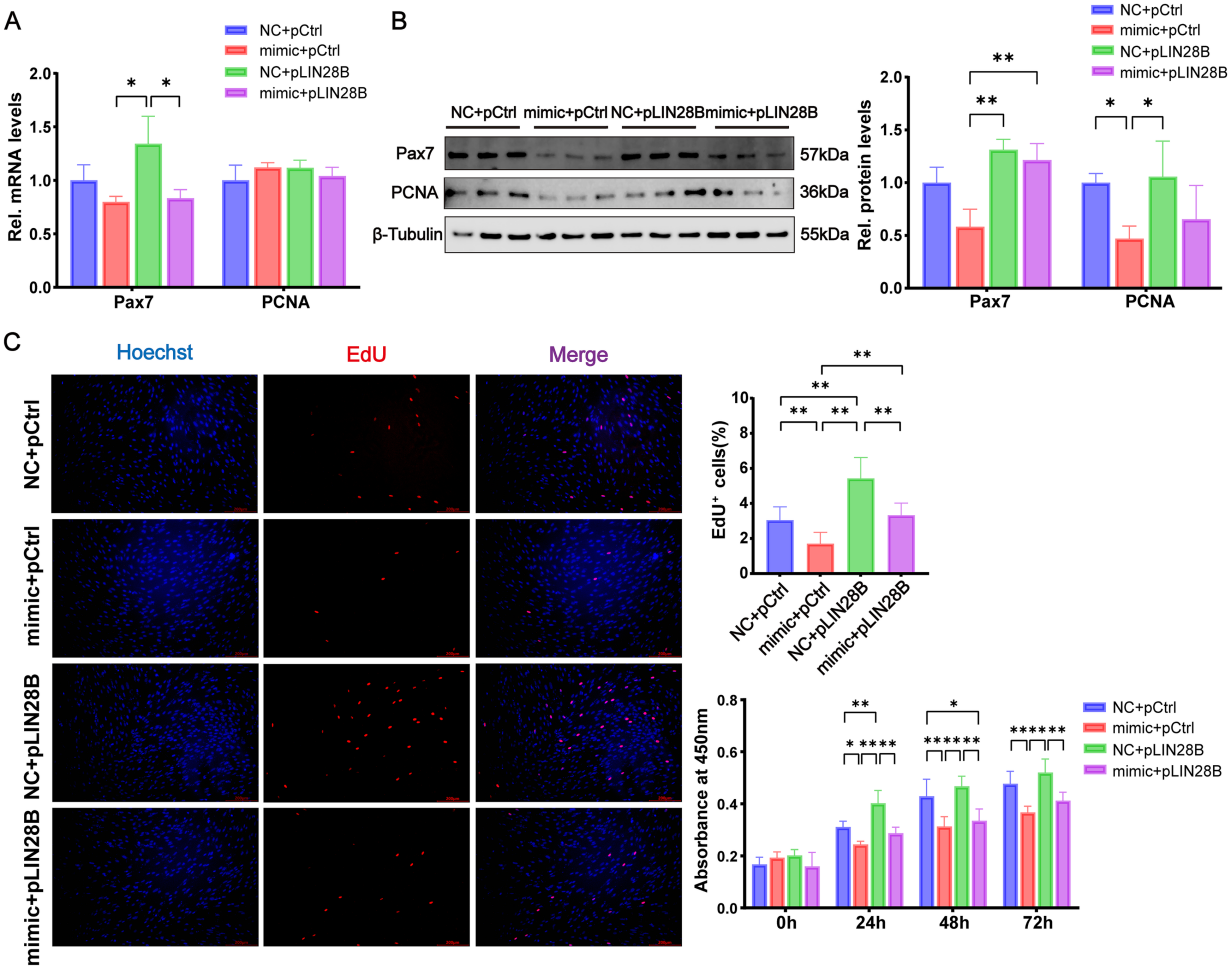
miR-379-5p suppresses goat MuSC proliferation by targeting *LIN28B*. **(A)** Relative mRNA levels of *Pax7* and *PCNA* in goat MuSCs transfected with NC or miR-379-5p mimics in combination with pCtrl or pLIN28B during the proliferation phase. **(B)** Western blot analysis of Pax7 and PCNA protein levels in proliferating MuSCs transfected with NC or miR-379-5p mimics in combination with pCtrl or pLIN28B. β-Tubulin was used as a loading control. **(C)** EdU and CCK-8 assays of goat MuSCs transfected with NC or miR-379-5p mimic in combination with pCtrl or pLIN28B, including representative immunofluorescence images and EdU^⁺^ cell quantification at 48 h post-transfection, as well as cell viability measured by CCK-8 at 0, 24, 48, and 72 h post-transfection. Scale bar = 200 μm. Each experiment contained three biological replicates. * *p* < 0.05, ** *p* < 0.01.

To determine whether miR-379-5p regulates MuSC proliferation through *LIN28B*, cells were subjected to four treatment conditions: NC + pCtrl, mimic + pCtrl, NC + pLIN28B, and mimic + pLIN28B. At the mRNA level, *Pax7* showed minor changes among groups, with a significant increase only in the NC + pLIN28B group compared with those in the mimic + pCtrl and mimic + pLIN28B groups (*p* < 0.05), while *PCNA* expression remained unchanged (Figure 6A). At the protein level, PCNA was significantly reduced in the mimic + pCtrl group compared with the NC + pCtrl and NC + pLIN28B groups (*p* < 0.05, Figure 6B). Similarly, Pax7 protein expression was significantly lower in the mimic + pCtrl group than in the NC + pLIN28B and mimic + pLIN28B groups (*p* < 0.01), although no difference was observed between the mimic + pCtrl and NC + pCtrl groups (Figure 6B).

Cell proliferation was further evaluated using EdU and CCK-8 assays (Figure 6C). The mimic + pCtrl group exhibited a significant reduction in EdU^+^ cells (*p* < 0.01) compared with the NC + pCtrl group, whereas *LIN28B* overexpression markedly increased EdU^+^ cells (*p* < 0.01). In the mimic + pLIN28B co-transfection group, the proportion of EdU^+^ cells was significantly higher than that in the mimic + pCtrl group (*p* < 0.01) but considerably lower than in the NC + pLIN28B group (*p* < 0.01). Consistent results were obtained with the CCK-8 assay. Together, these results suggest that miR-379-5p suppresses the proliferative capacity of goat MuSCs by targeting *LIN28B*.

### 3.7 miR-379-5p inhibits differentiation and promotes mitochondrial function of goat MuSCs by regulating *LIN28B*

**Figure 7:**
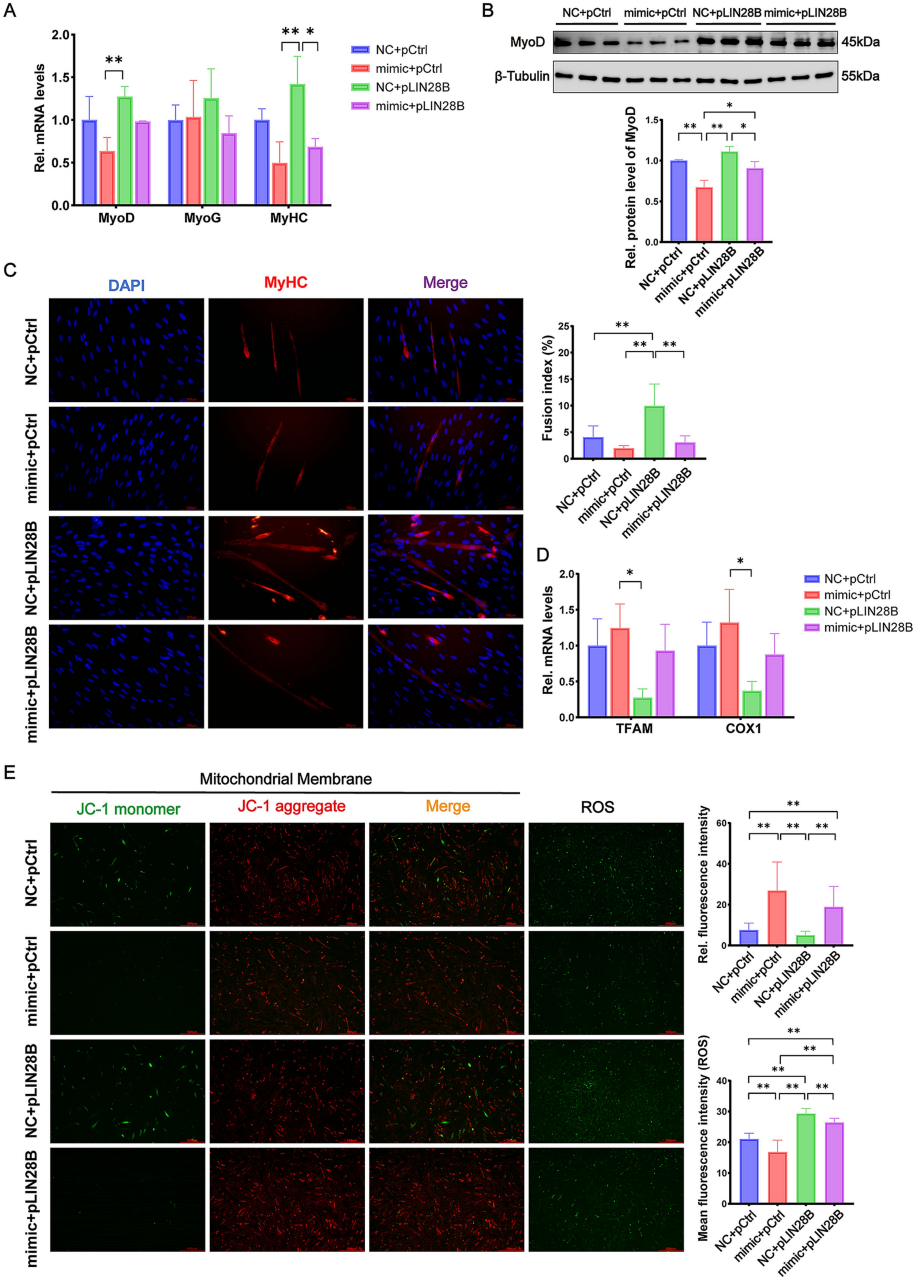
miR-379-5p inhibits goat MuSC differentiation and improves mitochondrial function by targeting *LIN28B*. **(A)** Relative mRNA levels of *MyoD*, *MyoG*, and *MyHC* in goat MuSCs transfected with NC or miR-379-5p mimics in combination with pCtrl or pLIN28B during the differentiation phase. **(B)** Western blot analysis of MyoD protein levels in differentiating MuSCs transfected with NC or miR-379-5p mimics in combination with pCtrl or pLIN28B. β-Tubulin was used as a loading control. **(C)** Immunofluorescence staining of MyHC and analysis of myotube fusion index in differentiating goat MuSCs. Scale bar = 100 μm. **(D)** Relative mRNA levels of mitochondrial marker genes (*TFAM* and *COX1*) in differentiating MuSCs transfected with NC or miR-379-5p mimics in combination with pCtrl or pLIN28B. **(E)** Representative fluorescence images illustrating mitochondrial membrane potential and ROS levels in differentiating MuSCs. Left: JC-1 staining indicates mitochondrial membrane potential. ROS production is indicated by green fluorescence. Middle: Ratio of fluorescence intensity. Right: Quantification of ROS fluorescence intensity. Scale bar = 500 μm. Each experiment contained three biological replicates. * *p* < 0.05, ** *p* < 0.01.

We next examined whether miR-379-5p regulates MuSC differentiation via *LIN28B*. According to Figure 7A, the mRNA levels of *MyoD* and *MyHC* were significantly upregulated in the NC + pLIN28B group compared with the mimic + pCtrl group (*p* < 0.01), whereas no significant changes were detected in *MyoG* expression (Figure 7A). Consistently, MyoD protein levels were markedly reduced by miR-379-5p overexpression and partially restored by *LIN28B* (*p* < 0.05, Figure 7B). MyHC immunofluorescence staining further confirmed these effects, showing that *LIN28B* significantly enhanced myotube formation, whereas miR-379-5p overexpression was associated with a non-significant reduction in the fusion index (*p* > 0.05). Notably, co-transfection attenuated the differentiation-promoting effect of *LIN28B* (Figure 7C). Collectively, these findings indicate that miR-379-5p suppresses differentiation of goat MuSCs by inhibiting *LIN28B* expression.

To verify whether these effects involved changes in mitochondrial function, we further assessed mitochondrial characteristics in co-transfected MuSCs. According to Figure 7D, the mRNA levels of *TFAM* and *COX1* in the mimic + pLIN28B group were between those of the NC + pLIN28B group and the mimic + pCtrl group, although the differences were not statistically significant (*p* > 0.05). Compared with the NC + pLIN28B group, miR-379-5p overexpression significantly upregulated the mRNA levels of *TFAM* and *COX1* (*p* < 0.05). JC-1 staining revealed that co-transfection with miR-379-5p and *LIN28B* significantly elevated the aggregate-to-monomer fluorescence ratio relative to the NC + pLIN28B group (*p* < 0.01), indicating improved mitochondrial membrane potential (Figure 7E). Additionally, ROS levels in the mimic + pLIN28B group were significantly lower than those in the NC + pLIN28B group (*p* < 0.01) but significantly higher than in the mimic + pCtrl group (*p* < 0.01; Figure 7E). Overall, these findings indicate that miR-379-5p mitigates the inhibitory effects of *LIN28B* on mitochondrial function during MuSC differentiation.

## 4 Discussion

miRNAs serve as critical post-transcriptional regulators in skeletal muscle development. Several conserved miRNAs, including miR-143 and miR-381, have been shown to modulate proliferation and differentiation of muscle cells (38). However, the functional roles of many miRNAs in satellite cell biology, particularly in livestock, remain uncharacterized. In goat MuSCs, miR-193b-3p has been shown to promote proliferation through *IGF2BP1* (22), whereas miR-27b inhibits proliferation but enhances differentiation by targeting *Pax3* (24). Transcriptome profiling has revealed that miR-379-5p is highly expressed in goat skeletal muscle (39), suggesting its potential involvement in myogenesis. Functional analyses revealed that miR-379-5p overexpression significantly inhibited both proliferation and differentiation of goat MuSCs, as evidenced by the downregulation of proliferation-associated genes (*Pax7*, *CCND1*, and *CDK2*), a lower proportion of EdU^+^ cells, and reduced expression of differentiation markers (*MyoD*, *MyoG*, and *MyHC*). These results are consistent with previous findings reporting that miR-379-5p inhibits cell proliferation in human breast cancer cells (40) and suppresses myogenic differentiation in mouse MuSCs (41). In our study, overexpression of miR-379-5p reduced mitochondrial membrane potential and elevated ROS levels during MuSC proliferation, whereas the opposite effects were observed during differentiation. A decrease in mitochondrial membrane potential is considered an early indicator of mitochondrial stress (42), while increased ROS production is often associated with mitochondrial dysfunction, mutations, or impaired OXPHOS (43). These stage-dependent changes in mitochondrial membrane potential and ROS align with the metabolic shift from glycolysis in proliferating MuSCs to OXPHOS during differentiation (44, 45).

We identified a conserved miR-379-5p binding site in the 3′UTR of goat *LIN28B*, consistent with previous reports in human cells (46, 47). This interaction was validated by dual-luciferase assays, and overexpression of miR-379-5p consistently led to a reduction in *LIN28B* mRNA levels during both proliferation and differentiation. *LIN28B* is an evolutionarily conserved mRNA-binding protein that promotes cell-cycle progression and lineage determination (28), and has been implicated in neuronal and germ cell development across various cell types (48, 49). In line with these established roles, our study revealed that *LIN28B* overexpression enhances both proliferation and differentiation capacities in goat MuSCs. Increasing evidence suggests that *LIN28B* participates in the regulation of mitochondrial metabolism, primarily through modulating pathways related to OXPHOS (50). Consistent with previous findings, our results indicate that *LIN28B* overexpression negatively affects mitochondrial function in goat MuSCs by reducing mitochondrial membrane potential and elevating ROS levels during both proliferation and differentiation phases.

In the current experiment, overexpression of miR-379-5p suppressed *LIN28B*-induced proliferation and differentiation in goat MuSCs, supporting the existence of a functional miR-379-5p/*LIN28B* regulatory axis in skeletal muscle development. This is consistent with previous studies in breast cancer and diabetic nephropathy models, where miR-379-5p was shown to suppress *LIN28B* expression and inhibit cell proliferation (46, 47). Furthermore, miR-379-5p co-transfection mitigated the decline in mitochondrial membrane potential and increase in ROS levels caused by *LIN28B* overexpression during differentiation, indicating that miR-379-5p attenuates *LIN28B*-induced mitochondrial dysfunction. These findings suggest that miR-379-5p regulates myogenic progression via *LIN28B* suppression and contributes to mitochondrial homeostasis during differentiation. Overall, these results provide new mechanistic insights into skeletal muscle development in goats and highlight miR-379-5p as a potential target for improving muscle growth and function.

## 5 Conclusion

In conclusion, miR-379-5p overexpression inhibited the proliferation and differentiation of goat MuSCs by targeting *LIN28B*, whereas *LIN28B* overexpression promoted these processes. Both miR-379-5p and *LIN28B* suppressed mitochondrial activity during the proliferative phase. However, in differentiating MuSCs, miR-379-5p overexpression attenuated *LIN28B*-mediated suppression of mitochondrial function, suggesting a critical miR-379-5p–*LIN28B* regulatory axis that modulates MuSC proliferation, differentiation, and mitochondrial homeostasis.

## Data Availability

The original contributions presented in the study are included in the article/Supplementary material, further inquiries can be directed to the corresponding author.

## Footnotes

1
https://www.targetscan.org/vert_80/

2
https://mirdb.org/cgi-bin/search.cgi

3
https://mirmap.ezlab.org/app

4
http://129.206.7.150/

5
https://www.mirbase.org/
