# Supplementary material for: miR-379-5p retards the proliferation and differentiation of goat skeletal muscle satellite cells by targeting LIN28B
Source: Front Vet Sci. 2025 Dec 4;12:1694160. doi: 10.3389/fvets.2025.1694160 (PMC12711551; doi:10.3389/fvets.2025.1694160)
Supplement: Supplementary file 1 [file Data_Sheet_1.zip › supplement table/supplement table S1.docx]

**Table S1** Primer sequences information

| Primer | Sequence of primer  (5’-3’) | Tm  (℃) | Length  (bp) |
| --- | --- | --- | --- |
| GAPDH | F: GCAAGTTCCACGGCACAG  R: GGTTCACGCCCATCACAA | 59 | 249 |
| MyoD | F:GTGCAAACGCAAGACGACTA  R:GCTGGTTTGGGTTGCTAGAC | 60.7 | 128 |
| MyoG | F:GGACCCTACAGATGCCCACAA  R:TTGGTATGGTTTCATCTGGG | 61.8 | 101 |
| MyHC | F:CCACATCTTCTCCATCTCTG  R:GGTTCCTCCTTCTTCTTCTC | 55 | 171 |
| MEF2C | F:ATCCTGATGCAGACGATTCAG  R:GGTGGAACAGCACACAATCTT | 59 | 115 |
| Myf5 | F:CACAACCAACCCTAACCA  R:TTGATCCGATCCACTATG | 54.3 | 315 |
| PCNA | F:CGCTTAAGGATCTCATCAATGAG  R:GTTACGGTCGCAGCGGTAAG | 61.4 | 143 |
| Pax7 | F:AGGACGAAGCGGACAAGAA  R:TCCAGACGGTTCCCTTTGT | 59.7 | 92 |
| CCND1 | F:CGTCCATGCGGAAGATCGT  R: ACAGGAAGCGGTCCAGGTAGT | 62.9 | 108 |
| CDK2 | F:CCGTCCACATAGGTTTCCCA  R:GTTGGCCTTACACAGTGGCT | 60.3 | 109 |
| LIN28B | F:CAGGTTTTGCACGGAACTGG  R:GGCTTCCCTCTCGGCTTATC | 55 | 88 |
| miR-379-5p | F:UGGUAGACUAUGGAACGUAGG | 54.3 | -- |
| COX1 | F:CCTCCTTTCACTTCCTGTATT  R:TGTGTTTAGGTTTCGGTCTGT | 53.7 | 67 |
| COX2 | F:TAGAGGTAGATAACCGAGT  R:GAACGATTGGTATGAAAC | 49.5 | 225 |
| CYCS | F:TGTTTGGAGAGGGGTAGGCT  R:CCAGTCCAGATACTTGCCCC | 56.9 | 101 |
| ATP5a1 | F:GTCTGTGTCCCGTGTTGGAT  R:ACTCAGTCAAACGCACACCA | 57.2 | 173 |
| TFAM | F:TAGTAGGTTGGGCAGGCTCT  R:CATCGCATCCGCAGACAAAG | 59.9 | 115 |

*: The miR-379-5p reverse primer was provided by the Takara miRNA reverse transcription kit.
